# CT genotype of 5,10-methylenetetrahydrofolate reductase (MTHFR) C677T polymorphism is protector factor of major depressive disorder in the Tunisian population: a case control study

**DOI:** 10.1186/s12991-016-0103-5

**Published:** 2016-07-30

**Authors:** Mohamed Amine Sayadi, Ons Achour, Asma Ezzaher, Ilham Hellara, Asma Omezzine, Wahiba Douki, Ali Bousslama, Lotfi Gaha, Mohamed Fadhel Najjar

**Affiliations:** 1 Laboratory of Biochemistry-Toxicology, Monastir University Hospital, Monastir, Tunisia; 2 Research Laboratory “Vulnerability to Psychotic disorders LR 05 ES 10”, Department of Psychiatry, Monastir University Hospital, Monastir, Tunisia; 3 LR 12 SP 11, Biochemistry Department, Sahloul Sousse University Hospital, Sousse, Tunisia

## Abstract

**Background:**

Major depressive disorder (MDD) is a common psychiatric disorder with considerable mortality. Death from unnatural causes, largely suicidal or quasi-suicidal, has a particularly high risk for the functional disorders, especially depression and schizophrenia. One of the prospective risk factors for this disease is hyperhomocysteinemia and folate deficiency. The methylenetetrahydrofolate reductase (MTHFR) gene encodes for a 5-methylenetetrahydrofolate reductase involved in folate metabolism and neurotransmitter synthesis. The aim of this research is to study the association between the C677T polymorphism of MTHFR gene and depression in Tunisian population, to explore their relationship with clinical and therapeutic characteristics of this disease. And it may lead to discover a novel marker to identify a patient with a higher risk of development of depressive disorder to be. This marker can be used for better therapeutic management and prevent disease installation.

**Methods:**

Our study included 208 depressive patients, 187 controls aged between 44.1 ± 13.5 and 38.9 ± 13.2 years, respectively. MTHFR gene polymorphisms were determined by PCR–RFLP (polymerase chain reaction–restriction fragment length polymorphism).

**Results:**

No significant difference was detected in the distribution of the genotype frequencies of MTHFR C677T polymorphisms (*χ*^2^ = 5.443, df = 2, *p* = 0.066) between patients and controls. But when we study the risk of these genotypes, CT genotype is significantly more frequent in controls compared to patients, it may be a protection from depression (OR = 0.655, CI 95 % = 0.432–0.995, *p* = 0.047, OR* = 0.638, CI 95 %* = 0.415–0.983, p* = 0.04, before and after adjustment). Women, TT Genotype can increase four times the risk to be depressive. Addictive behavior seems to be associated with CT genotype and there was no significant association between clinical and therapeutic characteristics and this polymorphism.

**Conclusion:**

This paper is the first study to prove that CT genotype of MTHFR C677T polymorphism may protect from depression and TT genotype seems to be associated with women’s depression. Further studies are required with other polymorphisms and biochemical factors that must be investigated to clarify the implication of MTHFR C677T polymorphism in the pathophysiology of depression.

## Background

Major depressive disorder (MDD) is a common psychiatric disorder with considerable mortality [1] which affects 121 million people worldwide [2]. The prevalence of depression in Tunisia is over 7 % [3]. Death from unnatural causes, largely suicidal or quasi-suicidal, has a particularly, high risk for substance abuse and eating disorders and a high risk for the functional disorders, especially MDD and schizophrenia [1]. The etiology of this MDD remains unclear, both gene–gene and gene-environment interactions are believed to have an important role on the development of this disease [4].

The genetic of depression has been extensively investigated, López-León et al. [5] has listed more than 393 polymorphisms in 102 genes, who were tested to have a possible relationship with depression. One promising candidate genetic marker for depression prognosis is the methylenetetrahydrofolate reductase (MTHFR) gene. This gene encodes for a 5-methylenetetrahydrofolate reductase involved in folate metabolism and neurotransmitter synthesis. Folate, vitamin B6 and vitamin B12 are used as cofactors in the metabolisation of homocysteine. Hyperhomocysteinemia and folate deficiency are prospective risk factors for depression [6–9]. Homocysteine or its metabolites may have an excitotoxic effect on neurons and may inhibit methylation processes in the central nervous system, it is toxic to the neurons, endothelial cells, can induce DNA strand breakage, oxidative stress, and apoptosis [10, 11]. This reduction of the MTHFR enzyme activity and the mild elevation of homocysteine plasma are probably caused by the C-T substitution at the 677 nucleotide in the MTHFR gene and it seems to be the major genetic cause [12]. Studies are focused on this hypothesis but controversial results are found in the case–control studies investigating the association between the MTHFR C677T polymorphism and depression. Gilbody et al. [7] and Wu et al. [13], in their meta-analysis have found a positive association with MTHFR C766T polymorphism and the risk of depression, but Zintzaras [14] and Gaysina et al. [15] reports the absence of association in their meta-analysis. These controversial results may be explained by the size of population and the ethnic and geographic variation between populations.

In our case, we searched to study the relationship of this polymorphism with depression and different characteristics (socio-demographic, clinical and therapeutics) in Tunisian population of center. And this may lead to discover a novel marker to identify a patient with a higher risk of development of depressive disorder. This genetic marker can be used for better therapeutic management and prevent disease installation through treatment, psychological support and nutrition. A detection particular combination of polymorphism of different genes may confer a better exploration of earlier onset or presence of the different forms of pathology.

## Patients and methods

### Study population

The study was conducted in the Department of Psychiatry, of the University Hospital of Monastir, Tunisia, included 208 depressive patients and 187 controls without psychiatric or somatic pathology. The mean age of the 208 patients, including 91 males and 117 females, was 44.11 ± 13.52 years, while the mean age of the 187 controls including 91 males and 95 females, was 38.53 ± 13.22 years.

This study was approved by the local ethical committee and all subjects were of Tunisian origin. It is interesting to note that the Tunisian population is essentially Arabs, Berbers or most of the time, mixed between those two ethnical origins [16]. Consensus on the diagnosis, according to the Diagnostic and Statistical Manual of Mental Disorders, fourth edition (DSM-IV) criteria (American Psychiatric Association, 2004), was made by psychiatrists. The exclusion criteria were age below 18 years, other psychiatric illnesses, epilepsy or mental retardation. All subjects were questioned about their age, gender, previous treatments, cigarette and alcohol consumption habits. All Controls filled out the same questionnaire and confirmed to be not depressed by the Beck Depression Inventory (BDI). The clinical and socio-demographic characteristics are shown in Table 1.

**Table 1:** Demographic and clinical data of the study population

	Patients	Controls	*p*
Gender: male/female (ratio)	91/117	91/96	0.328
Age (years)	44.11 ± 13.518	38.53 ± 13.222	<0.001
Age group			
Youth (<20 years)	11 (5.3 %)	17 (9 %)	0.021
Adult (20 ≤ years < 50)	122 (58.6 %)	125 (66.9 %)	
Old (≤50 years)	75 (36.1 %)	45 (24.1 %)	
BMI (kg/m^2^)	27.098 ± 5.60	26.962 ± 4.58	0.793
Obesity			
Normal	72 (34.6 %)	71 (38 %)	0.361
Over weight	75 (36.1 %)	73 (39 %)	
Obese	61 (29.3 %)	43 (23 %)	
Cigarette smoking			0.93
Smokers	64 (32 %)	59 (32 %)	
Non-smokers	136 (68 %)	128 (68 %)	
Alcoholic beverages			0.807
Consumers	42 (20.2 %)	40 (21.4 %)	
Non-consumers	164 (78.8 %)	147 (78.6)	
Drugs use			
Yes	17 (8.2 %)	8 (4.3 %)	0.112
No	191 (91.8 %)	179 (95.7 %)	
Treatments			
No	26	–	–
Tricyclic antidepressants (TCA)	42	–	
SIRS	94	–	
Atypique	16	–	
SIRS + TCA	6	–	

### Genotype analysis

Genomic DNA was extracted and isolated from EDTA blood samples by salting-out method [17]. The MTHFR C677T polymorphisms of the 5,10-methylenetetrahydrofolate reductase gene has been studied by the protocol described previously by Frost et al. [18]. In brief, a polymerase chain reaction (PCR) method using specific primers was carried out and the PCR products were digested with *Hin*fI and then electrophoresed on 3 % agarose gels stained with ethidium bromide and UV transillumination. The uncut amplicon is 198 base pairs (bp) length. After digestion, the “T” allele gives 173 and 25 bp fragments, and the “C” allele gives one fragment with 198 bp fragments.

### Statistical analysis

We have performed a case–control study on depressive and safe Tunisian population. Statistical analyses were performed using SPSS 17.0 (SPSS, Chicago, IL, USA). Hardy–Weinberg equilibrium was evaluated and genotype frequencies were compared by Chi-squared test (*χ*^2^). Quantitative variables were presented as mean ± SD and comparisons were performed using the Student’s *t* test. Qualitative variable comparisons were performed using the *χ*^2^ test. Odd ratios (ORs) and their 95 % confidence interval (CI) were calculated. The differences in demographic, clinical and therapeutic characteristics of patients were assessed among the MTHFR genotype using analysis of variance (ANOVA). Adjustment for potential confounder factors (age, drugs consumption) was performed by binary logistic regression. The statistical significance level was set at *p* ≤ 0.05.

## Results

In our population, depression is highly associated with age (*p* < 0.001) and specifically with old persons (≤50 years) with *p* = 0.021, but no difference has been associated with the other demographic characteristics of our population (Table 1). Moreover, no significant difference was detected in the genotype frequencies (*χ*^2^ = 5.443, df = 2, *p* = 0.066) between patients and controls (Table 2).

**Table 2:** Genotype frequencies of the C677T polymorphisms in the gene in patients and in controls

Genotype	Patients	Controls	*χ* ^2^, df, *p*
CC	105 (51 %)	80 (43 %)	*χ* ^2^ = 5.443/ddl = 2//*p* = 0.066
CT	80 (38 %)	93 (50 %)	
TT	23 (11 %)	14 (7 %)	

On the other side, study of risk demonstrated that CT genotype was more frequent in controls then depressives (51 vs 43 %, respectively), with significant association (OR = 0.655, CI 95 % = 0.432–0.995, *p* = 0.047, OR* = 0.638, CI 95 %* = 0.415–0.983, p* = 0.041 before and after adjustment to confound factors) (Table 3). However, TT genotype was more prevalent in depressive (11 %) compared to controls (7 %), but not significant (*p* = 0.544 and p* = 0.698 before and after adjustment) (Table 3). When we focused on gender, we have found a positive association between TT genotype and the pathology in Women (OR = 4.161, CI 95 % = 0.886–19.540, *p* = 0.053, OR* = 0.4.233, CI 95 %* = 0.834–21.391, p* = 0.082), but no association was found for men (Tables 4, 5, 6, 7).

**Table 3:** Association between depression and MTHFR C766T polymorphisms

	OR	IC 95 %	*p*	ORa	IC 95 % adjusted	*p*
N(CC)	1	–	–			
H(CT)	0.655	0.432–0.995	0.047	0.638	0.415–0.983	0.041
M(TT)	1.252	0.606–2.585	0.544	1.161	0.546–2.469	0.698

**Table 4:** Genotype frequencies of the C677T polymorphisms in the gene in men patients and in men controls

	Patient	Controls	Total	
MTHFR1				
N(CC)	43	37	80	*χ* ^2^ = 0.810/ddl = 2//*p* = 0.667
H(CT)	37	42	79	
M(TT)	11	12	23	
Total	91	91

**Table 5:** Association between depression and MTHFR C766T polymorphisms in men

	OR	IC 95 %	*p*	ORa	IC 95 % adjusted	*p*
N(CC)	1	–	–	–	–	–
H(CT)	0.758	0.406–1.414	0.383	0.645	0.333–1.250	0.194
M(TT)	0.789	0.312–1.997	0.616	0.755	0.295–1.933	0.558

**Table 6:** Genotype frequencies of the C677T polymorphisms in the gene in women patients and in women controls

	Patients	Témoin	Total	
MTHFR1				
N(CC)	62	43	105	*χ* ^2^ = 8.921/ddl = 2//*p* = 0.012
H(CT)	43	50	93	
M(TT)	12	2	14	
Total	117	95	212

**Table 7:** Association between depression and MTHFR C766T polymorphism in women

	OR	IC 95 %	*p*	ORa	IC 95 % adjusted	*p*
N(CC)	1	–	–	–	–	–
H(CT)	0.596	0.340–1.048	0.071	0.613	0.342–1.099	0.100
M(TT)	4.161	0.886–19.540	0.053	4.233	0.834–21.391	0.082

In addictive behavior, CT genotype was present frequently in our depressive population. 61.9 % of alcohol consumers have the CT genotype, compared to no consumers who have just 35.6 % with (*p* = 0.001). In the other side, the CT genotype was present in 70.6 % of the depressive consumers of drugs of abuse (*p* = 0.013) (Table 8). We also found that 50 % of smokers have the CT genotype (*p* = 0.058).

**Table 8:** Demographic, clinical and therapeutic characteristics of Depressives patients according to the MTHFR C766T genotypes

	MTHFR			*p*
	CC	CT	TT	
Age	45.37 ± 13.036	42.16 ± 14.132	45.09 ± 13.287	0.261
BMI	27.127 ± 5.992	26.8113 ± 5.992	27.965 ± 5.201	0.685
Gender				
Man	43 (47.2 %)	37 (40.7 %)	11 (12.1 %)	0.707
Women	62 (53 %)	43 (36.8 %)	12 (10.2 %)	
Alcohol				
Yes	12 (28.6 %)	26 (61.9 %)	4 (9.5 %)	0.001
No	19 (11.6)	52 (31.7 %)	93 (56.7 %)	
Drugs				
Yes	5 (29.4 %)	12 (70.6 %)	(0 %)0	0.013
No	100 (52.4 %)	68 (35.6 %)	23 (12 %)	
Smoker				
Yes	28 (41.2 %)	34 (50 %)	6 (8.8 %)	0.058
No	77 (55 %)	46 (32.9 %)	17 (12.1 %)	
Suicide attempts				
Yes	9 (32.1 %)	14 (50 %)	5 (17.9 %)	0.099
No	96 (53.3 %)	66 (36.7)	18 (10 %)	
Characteristics				
Psychotic	19 (45.2 %)	16 (38.1 %)	7 (16.7 %)	0.705
Melancholic	71 (51.1 %)	55 (39.6 %)	13 (9.3 %)	
Atypical	15 (55.6 %)	9 (33.3 %)	3 (11.1 %)	
Severity 2				
Low	12 (36.4 %)	14 (42.4 %)	7 (21.2 %)	0.052
Medium	75 (55.1 %)	52 (38.2 %)	9 (6.7 %)	
Severe	18 (46.2 %)	14 (35.9 %)	7 (17.9 %)	
Anxiety associated				
Yes	60 (54.1)	39 (35.1)	12 (10.8)	0.522
No	45 (46.4)	41 (42.3)	11 (11.3)	
Type of anxiety				
Panic	23 (67.7 %)	10 (29.4)	1 (2.9 %)	0.129
General anxiety	37 (48.1 %)	30 (38.9 %)	10 (13 %)	
Obsessive compulsive	4 (100 %)	0	0	
Panic and general anxiety	0	1 (100 %)	0	
Personality disorders				
No	39 (50 %)	32 (41.1 %)	7 (8.9 %)	0.702
Yes	66 (50.8 %)	48 (36.9 %)	16 (12.3 %)	
Cluster				
Cluster A	1 (25 %)	2 (50 %)	1 (25 %)	0.818
Cluster B	18 (48.7 %)	15 (40.5 %)	4 (10.8 %)	
Cluster C	10 (55.6 %)	6 (33.3)	2 (11.1 %)	
Adaptation				
No	27 (54 %)	19 (38 %)	4 (8 %)	0.662
Almost	44 (45.4 %)	40 (41.2 %)	13 (13.4 %)	
Better	34 (55.8 %)	21 (34.4 %)	6 (9.8 %)	
Treatment				
No	28 (54.9 %)	18 (35.3 %)	5 (9.8 %)	0.806
TCA	19 (45.2 %)	16 (38.1 %)	7 (16.7 %)	
SIRS	48 (51.1 %)	37 (39.3 %)	9 (9.6 %)	
Atypical	8 (50 %)	6 (37.5 %)	2 (12.5 %)	
TCA + SIRS	2 (33.3 %)	4 (66.7 %)	0	
Antidepressor				
Amitriptyline	10 (40 %)	11 (44 %)	4 (16 %)	0.557
Clomipramine	11 (52.4 %)	7 (33.3 %)	3 (14.3 %)	
Escitalopram	1 (14.3 %)	4 (57.1 %)	2 (28.6 %)	
Fluoxétine	38 (56.7 %)	23 (34.3 %)	6 (9 %)	
Paroxétine	9 (56.3 %)	6 (37.5 %)	1 (6.2 %)	
Sertraline	2 (40 %)	3 (60 %)	0	

*SIRS* selective inhibitors of serotonin reuptake, *TCA* tricyclic antidepressant, *Atypical* venlafaxine, milnacipran

For those who have suicide attempts history, 17.9 % of them have the TT genotype, and 50 % have CT genotype (*p* = 0.099) (Table 8). Moreover, we noticed that CC genotype is more frequent then CT and TT genotypes in patients who have atypical depression (55.6 %) with medium severity (71.4 %) which associated to anxiety (54.1 %) specially panic one (67.7 %). In addition, we found that CC genotype is also more frequent with patients who have depression Cluster C (Table 8). Depressive patients, who have CT genotype, are frequently treated by Mixed Antidepressant (selective inhibitors of serotonin reuptake, tricyclic antidepressant) and usually use sertraline (Table 8).

## Discussion

To our knowledge, no study was done in Tunisian depressed population related to the C677T polymorphism. A significant association was found between age and pathology, The patients have the highest mean age with 44.11 ± 13.52 years, and the pathology is associated with the oldest cohort of our population (>50 years) with *p* = 0.021. Elderly person have many difficulties with activities of daily living, and many studies demonstrated that this difficulties are associated with increase of depressive symptoms increased with age [19]. Our result can be explained also by the higher levels of homocysteine usually in depressed subjects compared to controls. The plasma level of homocysteine is influenced by factors such as age, so it is possible that such effects contribute to the cascade of events leading to depression in later life [20].

We have not found any association between MTHFR polymorphism and unipolar depression, this result is similar to the findings of Kunugi et al. [21] and Henriquez-Hernandez et al. [22]. In a cohort of 277 Slovak with 143 controls subjects and 134 depressed patients, Evinova et al. [4] did not find any significant association with this polymorphism and major depressive disorders. On the other hand, meta-analysis of 26 studies, conclude that the MTHFR C677T is associated with increased risk of depression [13]. Reif et al. [23], have found a positive association between the same MTHFR polymorphism and Major depressive disorder.

In our study, CT genotype was more frequent and significantly in controls than depressives and after adjustment, this association remained significant. So CT genotype may be protecting from depression. This result is annoyed by the work of Shen et al. [24], who examined 368 patients and 219 controls, and he found a positive association between the CT genotype (*p* < 0.001) and depression.

Arinami et al. [25] have been the first ones who focused on this gene polymorphism, and found that TT genotype has a higher prevalence with depression with 2.8 as odd ratio. The same result was found in the meta-analysis of 17 articles by Peerbooms et al. [26] suggesting that TT genotype increase risk of receiving the diagnosis of major depressive disorders.

On the other hand, Bjelland et al. [6], report a positive association between TT genotype and higher level of homocysteine with depression without comorbid anxiety disorder. In Slovak population, TT genotype of C677T and AC genotype of A1298C of BDNF gene are considerably associated with the higher risk of major depression disorders [4].

In Poland population, Chojnicka et al. [27], have not found any association between the polymorphism and completed suicide, the same result is found in our study. A lake of association is supported by several studies [21, 23, 28–31]. The same contradictory results are shown in meta-analysis, Gilbody et al. [7], Lok et al. [32], López-León et al. [5] and Wu et al. [13] indicate that TT genotype increase the risk of depression disorders but Gaysina et al. [15] and Zintzaras [14], confirmed a lake of signification between the polymorphism and the pathology.

These controversial results may be due to the small sample sizes or to the influence of ethnic and demographic differences. Wu et al. [13] found that MTHFR C677T polymorphism is correlated to depression of Asian population in all genotype models, but only a marginal correlation was observed among white populations in recessive model. He showed that Asian population had a greater genetic risk to develop depression in comparison with white population. This also can be explained by the nature of food which is fortified with folate, so the TT effect cannot be observed [33].

According to gender, no association was found in men between depression and C677T, but in women, we found a positive association (*χ*^2^ = 8.921, df = 2, *p* = 0.012) and that TT genotype increase the risk of developing depression by four times referring to CC genotype with (*p* = 0.053 and p* = 0.082 before and after adjustment). These results show a greater genetic risk in women to develop depression.

Słopien et al. [34] found a higher correlation with TT genotype and depression found in post menopausal women (OR = 3.478; 95 % CI = 1.377–8.783; *p* = 0.0096), and this genotype displayed about 4.8-fold increased risk of moderate and severe depression (CI 95 % = 1.975–11.820, *p* = 0.0008). This association is confirmed by the work of Lewis et al. [35] on British Women’s Heart and Health Study with 545 women (OR = 1.35, 95 % CI = 1.1–1.8). It’s not supported by Kempisty et al. [36] who found a higher percentage of T allele in men compared to women and suggest that the 667T allele might represent a minor liability in women or an increased survival advantage for men.

No association is found in the work of Gaysina et al. [15] between depressive women and C677T polymorphism with (*χ*^2^ = 2 1.5; ddl = 2; *p* = 0.47) but Almeida et al. [37] did not find association in older women. Focusing on demographic characters, we find that CT genotype seems to be associated with addictive behavior in depressed people [37].

In our population, the major frequency of alcohol consumers, drugs abuse and smokers has a CT genotype who has been demonstrated previously as a protector from depression. We can explain this result by the neutralization of the protective effect of the CT genotype by the addictive behavior and the gene environment interaction. Patients need a good lifestyle to be protected, without addictive behavior that can change the gene effect.

Otherwise these associations can be explained by the relation between the levels of homocysteinemia and C677T genotype. Hultberg [38] demonstrated that chronic alcoholism was associated with hyperhomocysteinemia. On the other side Benyamina et al. [39] suggest that MTHFR 677TT genotype could play a protective role against alcohol dependence.

Lok [40] suggest that the interaction between MTHFR C677T and cannabis increased risk of recurrence in recurrent major depressive disorder patients over 5.5 years of follow-up and is associated with depressive symptoms in the general population. The study of Guillem et al. [41] confirms the high mood and anxiety disorders comorbidity among dependent users of cannabis. Also there are also studies that is showing low folate level due to cannabis abuse in pregnant women [42] and undergraduate students [43]. Cannabis abuse is associated with higher Homocysteine, as well as lower HDL and vitamin B12 levels first-episode schizophrenia [44].

Smoking is also associated with depression, and smoking is known to be associated with higher homocysteine plasma levels [45]. Moreover, Brown [46] confirms that the T allele and smoking interact in their association with homocysteine plasma levels. The T allele in smoking group had the highest level of homocysteine, in the opposite of C allele who had the lowest levels of homocysteine plasma.

Variant alleles (CT, TT) have been linked with increased serum homocysteine concentrations [47, 48], so this association can be explained by the gene environment influence on homocysteine level and cause depression, and we think that drugs, alcohol and smoking intake interfere with folate metabolism and affect the level of homocysteinemia.

No significant association was found between rs1801133 (C677T) polymorphism and suicide behavior (*p* = 0.682), suicide ideation (*p* = 0.603) and suicide attempts (*p* = 0.629) [49]. In Polish Caucasians population, MTHFR C677T polymorphism is admitted like not a risk factor for completed suicide with TT [27].

No significant association was found between treatment and MTHFR C677T polymorphism, but CT genotype was more frequent in patient, who use of the mixed antidepressant. This choice by doctors can be explained by the responses of depressive patients to the treatment. In literature, Shen et al. [24] report in Chinese Han population that MTHFR C677T interact with COMT Val158Met, to increase Major depressive disorder but without influencing on treatment responses. Bousman et al. [49] show the absence of remission for patients who have CC genotype after treatment. This contradiction may be explained by the influence of environmental factors, genetic variant interaction and socio-demographical interaction.

## Conclusions

This paper is the first study to prove that in Tunisian population of center region exist positive associations between depressive disorder and the C677T polymorphism and especially with CT and TT genotype. In our population, the more a person becomes aged, the more he is risked to have this pathology. In our case of study, we found that TT genotype is associated with depression and this association is more frequent in women, more than in men. In another hand, we considered that CT genotype is a protector from depression; however, major frequency of alcohol consumers, drugs abuse and smoking patients has a CT genotype, so it is no longer a protector because it seems that it is associated with addictive behavior in depressed people.

So this genotype may be used as a genetic marker to identify a patient with a higher risk of development of depressive disorder for better therapeutic management and prevent disease installation through treatment, psychological support and nutrition. But this study must be confirmed with other works with larger population.

More genetic and biochemical investigations are recommended to have a better comprehension for this disease. A detection particular with combination of polymorphism of different genes may confer a better exploration of earlier onset or presence of the different forms of pathology. Further studies of genetic polymorphism and a possible correlation with pathology, may lead to discover a novel marker to identify a patient with a higher risk of development of depressive disorder.
